# Rosmarinic Acid Inhibits PRV Replication by Regulating Oxidative Stress Through the Nrf2 Signaling Pathway

**DOI:** 10.3390/ani16030493

**Published:** 2026-02-04

**Authors:** Ruifei Li, Yanfeng Zhang, Zhaokun Wan, Zhiyuan Ren, Zhiying Wang, Juanjuan Xu, Yan Zhu, Su Li

**Affiliations:** 1 College of Veterinary Medicine, Northeast Agricultural University, Harbin 150038, China; 15733469806@163.com (R.L.); yanfengzhang2023@163.com (Y.Z.); 13393986356@163.com (Z.W.); zhiyuanren0328@163.com (Z.R.); wzyzy0419@163.com (Z.W.); xu101066@163.com (J.X.); 2 State Key Laboratory for Animal Disease Control and Prevention, National African Swine Fever Para-Reference Laboratory, National High-Containment Facilities for Animal Disease Control and Prevention, Harbin Veterinary Research Institute, Chinese Academy of Agricultural Sciences, Harbin 150038, China

**Keywords:** PRV, rosmarinic acid, Nrf2 signaling pathway, in vivo and in vitro

## Abstract

Pseudorabies (PR) is an acute and highly contagious disease caused by the pseudorabies virus (PRV). This virus has a wide range of susceptible hosts and has caused major economic losses to the global swine industry. While rosmarinic acid possesses broad antioxidant and antiviral properties, its efficacy against PRV has remained unexplored. Therefore, this study aimed to evaluate the anti-PRV activity of rosmarinic acid and to elucidate its underlying mechanism, with a focus on the nuclear factor erythroid 2-related factor 2 (Nrf2) signaling pathway. The results revealed that rosmarinic acid exhibited potent, concentration-dependent antiviral activity in vitro, with a half-maximal inhibitory concentration (IC50) of 0.02654 mg/mL, a half-maximal cytotoxic concentration (CC50) of 0.1043 mg/mL, and a selectivity index (SI) of 3.9. Rosmarinic acid inhibited virus adsorption, entry, and intracellular replication. It also significantly suppressed the expression of the gB protein. In a mouse model, rosmarinic acid treatment (200 mg/kg) significantly enhanced the survival rate to 28.5%. This treatment reduced the viral load in the brain, lungs, kidneys, heart, and spleen. It also alleviated the tissue damage caused by PRV infection. Furthermore, rosmarinic acid counteracted PRV-induced oxidative stress by elevating the activity of the antioxidant factors SOD and CAT and reducing the level of the oxidative factor MDA. Network pharmacology analyses predicted the Nrf2 signaling pathway as a key target for rosmarinic acid. Subsequent mechanistic studies confirmed that rosmarinic acid upregulated the expression of the Nrf2, HO-1, GPX, SOD, and CAT genes, as well as Nrf2 and HO-1 proteins, thereby promoting the nuclear translocation of Nrf2. These results identify rosmarinic acid as a promising anti-PRV agent that acts through multi-phase viral inhibition and activation of the Nrf2-mediated antioxidant defense, suggesting its potential as a novel pharmacological strategy against PRV.

## Simple Summary

This study represents the first systematic evaluation of the efficacy of rosmarinic acid against pseudorabies virus. The findings reveal that rosmarinic acid demonstrates promising antiviral activity both in vitro and in vivo. It effectively inhibits viral entry and replication, significantly enhances the survival rate of infected mice, and mitigates virus-induced damage across multiple organs. Further mechanistic investigation indicates that rosmarinic acid functions by activating a crucial endogenous antioxidant defense system, namely the Nrf2 pathway. This research provides a novel scientific foundation and suggests potential directions for the development of new drugs or strategies to control pseudorabies.

## 1. Introduction

Pseudorabies virus (PRV) is a member of the Herpesviridae family and belongs to the genus Varicellovirus [1]. This virus is the causative agent of pseudorabies (PR), a disease that not only causes major economic losses to the swine industry but also severely impacts the health and welfare of animals. Since its first identification in the early 20th century [2], PRV has spread globally, particularly in regions with highly developed swine industries, resulting in incalculable economic losses. PRV infects its hosts through the respiratory and digestive tracts. After entering the host, the virus rapidly spreads to the central nervous system, which can lead to various clinical symptoms, including neurological abnormalities, respiratory distress, and reproductive issues [3]. Severe cases can result in the death of piglets [4]. Once a herd is infected with this virus, it is difficult to eliminate it from the population [5]. Additionally, human infection with PRV can produce conditions such as endophthalmitis and encephalitis and, in severe cases, can be fatal. Slaughterhouse and aquaculture workers are the groups most at risk of infection [6,7]. PRV has a complex genomic structure and diverse variants [8], which pose challenges to the efficacy of traditional vaccines [9]. Currently, effective vaccines and treatment methods targeting PRV are lacking, hindering our ability to control this disease.

Natural compounds generally have few side effects, as they are often derived from plants and other natural sources. These molecules are relatively more accessible and have advantages in drug development, health maintenance, disease prevention, and sustainable development [10]. Several plant-derived compounds have demonstrated promising activity against PRV. For example, luteolin, a natural flavonoid compound found in various plants, such as honeysuckle and chrysanthemum, can reduce the viral copy number by affecting the attachment, entry, and subsequent replication processes of PRV [11]. Piceatannol, a polyphenolic compound found in grapes, strawberries, and certain herbs, can reduce the transcriptional level of the PRV genome and inhibit PRV-induced apoptosis [12]. Emodin, a compound extracted primarily from rhubarb and other plants, can significantly increase the survival rate of mice infected with PRV, inhibit the replication of PRV in various organs of infected mice, and alleviate the tissue and organ damage caused by PRV infection [13]. These findings validate the pursuit of natural antivirals for PRV.

Rosmarinic acid is a naturally occurring polyphenolic compound that is widely found in rosemary, mint, and sage plants, among other plants. It has edible value. Rosmarinic acid has significant antioxidant [14], anti-inflammatory [15], neuroprotective, and antitumour [16] properties. This molecule can increase the activity of cellular antioxidant enzymes and effectively scavenge free radicals, thereby protecting cells from oxidative damage. In addition, some studies have shown that rosmarinic acid has inhibitory effects on certain viruses, including the dengue virus [17], Japanese encephalitis virus [18], and EV71 virus [19]. This study evaluated the in vitro and in vivo inhibitory effects of rosmarinic acid on PRV. Network pharmacology was used to predict and experimentally verify the potential targets and pathways involved. Our work identifies rosmarinic acid as a promising therapeutic candidate and unveils a novel mechanism that could inform the development of strategies to combat PRV infection.

## 2. Materials and Methods

### 2.1. Cells, Viruses and Reagents

PK15 (pig kidney) cells are preserved in this laboratory. PK-15 cells were cultured in DMEM supplemented with 10% foetal bovine serum and 1% penicillin/streptomycin. The culture medium used for cell cytotoxicity and antiviral assays contained 2% foetal bovine serum. The PRV TJ strain (GenBank: KJ789182.1) was maintained in our laboratory. The virus was propagated by serial passaging in PK-15 cells, and the 50% tissue culture infectious dose (TCID50) was calculated to be 106.94/0.1 mL via the Reed–Muench method. The virus stock was stored at −80 °C until use. Rosmarinic acid was purchased from Dalian Meilun Biotechnology Co., Ltd., Dalian, China, with a purity of 98%. This molecule was dissolved in 1% dimethyl sulfoxide (DMSO) for stock solution preparation.

### 2.2. Cytotoxicity and Inhibitory Activity Assays

PK-15 cells were seeded in a 96-well plate and cultured until they formed a monolayer. A series of twofold dilutions of rosmarinic acid (concentration range: 0.015625 to 2 mg/mL) were subsequently added to the cells, which were then coincubated for 48 h. A cell control group was also included. The CCK-8 solution (MA0218, Meilun, Dalian, China) was added according to the manufacturer’s instructions, and the samples were incubated at 37 °C for 55 min. The optical density (OD) values at 450 nm were then measured via a microplate reader. The cell viability was calculated to evaluate the cytotoxicity of rosmarinic acid. The samples were incubated at 37 °C for 1 h. The above mixture was added to a 96-well cell culture plate and incubated for 1 h. The cells were washed with PBS, and cell maintenance medium was added. After 48 h of treatment, CCK-8 solution was added to the wells. The plates were incubated at 37 °C for 55 min. The OD value of each well was measured at a wavelength of 450 nm. The viral inhibition rate was calculated to evaluate the inhibitory activity of rosmarinic acid against PRV. With GraphPad Prism 8.4 software, the half-maximal cytotoxic concentration (CC50) and half-maximal inhibitory concentration (IC50) of rosmarinic acid were calculated.

### 2.3. In Vitro Inhibition Assay

#### 2.3.1. Virus Adsorption Stage

PK-15 cells at 80–90% confluency were prechilled at 4 °C for 1 h. A 100 TCID50 PRV stock mixture and an equal volume of rosmarinic acid were mixed and incubated with the cells at 4 °C for 1 h. The supernatant was discarded, the mixture was washed twice, cell maintenance medium was added, and the mixture was cultured at 37 °C and 5% CO_2_. A virus control group was also established. The cell samples were collected 48 h later, and the viral genomic DNA was extracted. The viral DNA copy number in the infected cells was quantified via FQ–PCR.

#### 2.3.2. Viral Entry Stage

PK-15 cells at 80–90% confluency were prechilled at 4 °C for 1 h. The cells were infected with 100 TCID50 of PRV at 4 °C for 1 h. After the cells were washed with prechilled PBS, cell maintenance medium containing rosmarinic acid was added. The mixture was incubated at 37 °C for 1 h, after which the medium was replaced with maintenance medium. The mixture was incubated at 37 °C with 5% CO_2_ for further culture. A virus control group was also established. The cell samples were collected 48 h later, and the viral genomic DNA was extracted. The viral DNA copy number in the infected cells was quantified via FQ–PCR.

#### 2.3.3. Viral Replication Stage

PK-15 cells were infected with 100 TCID50 of PRV at 37 °C for 1 h. The virus-containing medium was removed, and the cells were washed twice. The cell maintenance medium was supplemented with rosmarinic acid. The mixture was incubated at 37 °C with 5% CO_2_ for further culture. A virus control group was also established. The cell samples were collected 48 h later, and the viral genomic DNA was extracted. The viral DNA copy number in the infected cells was quantified via FQ–PCR.

The viral genomic DNA was extracted via the TIANamp Genomic DNA Kit(TIANGEN; Beijing, China). The viral DNA copy number in the infected cells was analysed via real-time quantitative PCR (FQ-PCR). The forward primer sequence was 5′-GCCGAGTACGACCTCTGCC-3′, the reverse primer sequence was 5′-CGAGACGAACAGCAGCCG-3′, and the probe sequence was 5′-HEX-CCGCGTGCACCACGAAGCCT-BHQ1. A standard plasmid containing the gI gene was used to generate a standard curve. The FQ–PCR conditions were 95 °C predenaturation for 10 min, 94 °C denaturation for 35 s, and 60 °C annealing for 35 s. The reaction consisted of 40 cycles.

### 2.4. Effects of Rosmarinic Acid on the Expression of the gB Protein

When the PK-15 cells reached confluency, they were washed twice with PBS. An equal volume of a mixture containing 100 TCID50 of PRV that had been incubated at 37 °C for 1 h and 0.03125 mg/mL of rosmarinic acid was added. The solution was discarded after 1 h. After being washed with PBS, the cells were resuspended in cell maintenance medium and cultured further. After 36 h, the cells were lysed with protein extraction buffer. The harvested cell samples were used for total protein extraction. The proteins were separated by 10% SDS–PAGE and then transferred to a nitrocellulose (NC) membrane. The membrane was blocked at room temperature for 2 h with 5% nonfat milk. Then, antibodies against PRV gB (1:500; kindly provided by Prof. Su Li, Harbin, China) and β-actin(1:10,000; Bioss, Beijing, China) were added, and the mixtures were incubated together. The membrane was incubated overnight at 4 °C. After washing, the samples were incubated with an HRP-conjugated goat anti-mouse IgG secondary antibody(1:10,000; Proteintech, Wuhan, China) at room temperature for 1 h. The proteins were visualized via an enzymatic chemiluminescence (ECL) reagent (BL520A, Bio-sharp; Hefei, China). The signals were analysed via ImageJ-1.53 software (developed by the National Institutes of Health, Bethesda, MD, USA). The difference in the gB protein level between the samples was normalized to the expression level of the β-actin protein.

### 2.5. Molecular Docking

The three-dimensional structure of rosmarinic acid was retrieved from the PubChem database (http://pubchem.ncbi.nlm.nih.gov/) (accessed on 5 August 2025). Using OpenBabel-3.1.1, the 3D structure file was converted to PDB format. The gB protein (PDB ID: 5YS6) was obtained from the RCSB PDB database (http://www.rcsb.org/) (accessed on 5 August 2025) and used as the molecular-docking receptor. Protein preparation involved removal of water molecules and phosphate ions in PyMOL 2.6.0, and the processed structure was saved as a PDB file. Molecular docking between receptor and ligand was performed with AutoDockTools-1.5.7, with the docking center set to (87.286 Å, 56.664 Å, 22.83 Å) and the grid box dimensions set to 38 Å × 30 Å × 118 Å. The best docking pose was selected, and receptor–ligand interactions were visualized in Discovery Studio 2021 Client; further analysis and figure preparation were carried out in PyMOL.

### 2.6. In Vivo Anti-PRV Activity of Rosmarinic Acid

A total of 46 female Kunming mice weighing 20 ± 2 g were purchased from the Changsheng Experimental Animal Center. After a 1-week acclimation period for feeding in the experimental animal laboratory at Northeast Agricultural University, the mice were randomly divided into 5 groups. The groups included a normal control group (6 mice), a virus control group (10 mice), a high-dose rosmarinic acid group (200 mg/kg, 10 mice), a medium-dose rosmarinic acid group (100 mg/kg, 10 mice), and a low-dose rosmarinic acid group (50 mg/kg, 10 mice). For the rosmarinic acid treatment groups, the mice were administered rosmarinic acid by oral gavage. The treatments were administered once daily for 3 consecutive days, with the normal control group and virus control group receiving an equivalent volume of DMEM by gavage. On day 4, the virus control group and the rosmarinic acid treatment group were intramuscularly injected with 0.1 mL of 1000 TCID50 PRV solution for challenge, whereas the normal control group was intramuscularly injected with an equal volume of DMEM. At 2 h post-infection, the rosmarinic acid treatment groups received intragastric administration of rosmarinic acid at doses of 200 mg/kg, 100 mg/kg, or 50 mg/kg. The treatments were administered once daily for 4 consecutive days. The normal control group and the virus control group received the same volume of DMEM. The body weight, behaviour, and health status of the mice were recorded daily. On day 3 post-infection, 3 mice were randomly selected from each group for blood collection and necropsy. Pathological changes in the organs of the mice were observed, and heart, liver, lung, kidney, brain, and spleen tissues were collected. The remaining mice were monitored to determine the survival rate during the remainder of the experiment. The collected mouse organ tissues were homogenized in saline solution to extract DNA for determination of the viral load in the tissues. The other part was fixed in 4% paraformaldehyde, followed by tissue sectioning and histopathological examination. The collected blood samples were allowed to clot at 4 °C for 20 min. The samples were subsequently centrifuged at 5000 rpm for 10 min to obtain the serum, and the concentrations of the SOD, MDA, and CAT cytokines in the mouse serum were measured via enzyme-linked immunosorbent assay (ELISA) kits (Andy gene; Guangzhou, China).

### 2.7. Pharmacological Network Research on the Antioxidant Effects of Rosmarinic Acid

#### 2.7.1. Target Screening for Rosmarinic Acid

The PubChem database (https://pubchem.ncbi.nlm.nih.gov/) (accessed on 15 August 2025) was searched for “rosmarinic acid” to retrieve the chemical structure of rosmarinic acid, which was saved in SDF format. Next, the SDF file of rosmarinic acid was uploaded to the reverse pharmacophore matching database PharmMapper (https://www.lilab-ecust.cn/pharmmapper/) (accessed on 15 August 2025), and the term “druggable pharmacophore models” was selected following the database’s official instructions. The number of matching target sites was set to 1000, and the ranking of the top 1000 target sites corresponding to rosmarinic acid was obtained. After the target was identified, the UniProtKB search function in the UniProt database was used, and the protein name was entered. On the basis of the database search and conversion operations described above, the retrieved proteins were corrected to their official values.

#### 2.7.2. Screening of Antioxidant Targets

The GeneCards database (https://www.genecards.org/) (accessed on 18 August 2025) was searched via the keyword “antioxidant”, and the target data related to antioxidant activity were retrieved and filtered.

#### 2.7.3. Visualization Analysis of the Antioxidant Targets of Rosmarinic Acid

The Venny 2.1 online software tool platform was used to generate a Venn diagram for visualization of the antioxidant targets of rosmarinic acid.

#### 2.7.4. Construction of a Protein–Protein Interaction (PPI) Network

With the String database (https://www.string-db.org/) (accessed on 18 August 2025), the potential antioxidant targets of rosmarinic acid were imported, with the species selected as “Homo sapiens”. This procedure allowed the retrieval of the PPI relationships of the target proteins. Medium confidence (0.400) was selected for the minimum required interaction score, and a PPI network graph and a corresponding TSV file were exported. The TSV file was imported into Cytoscape 3.7.2 software to generate a PPI network. A topological analysis of the network was conducted via the Network Analyzer tool. The selection criterion was nodes with a degree greater than twice the median degree. The selection basis was nodes with a median betweenness centrality and closeness centrality. On the basis of these criteria, a HitHub network was generated to identify the core target nodes.

#### 2.7.5. Potential Pathways for Antioxidant Regulation by Rosmarinic Acid

For further elucidation of the potential pathways involved in the antioxidant effects of rosmarinic acid, the Metascape database was accessed, and the potential target genes involved in the antioxidant effects of rosmarinic acid were identified. Gene Ontology (GO) analysis and Kyoto Encyclopedia of Genes and Genomes (KEGG) pathway analysis were performed on the identified targets. The number of targets was sorted in descending order. The enriched GO terms included biological process (BP), cellular component (CC), and molecular function (MF) terms. The GO function and KEGG pathway analyses were visualized via a bioinformatic platform.

### 2.8. Effects of Rosmarinic Acid on the Nrf2 Signaling Pathway in PRV-Infected Cells

For analysis of the effect of rosmarinic acid on the Nrf2 pathway in PRV-infected cells, Western blotting and real-time fluorescent quantitative PCR were used to determine the expression levels of Nrf2, HO-1, and the downstream antioxidant genes GPX1, GPX2, SOD1, SOD2, NQO1, and CAT in the Nrf2 pathway. The nuclear translocation rate of the Nrf2 protein was determined via indirect immunofluorescence.

The Western blot procedure was performed as described in Section 2.5. The primary antibodies used were rabbit anti-Nrf2 antibody (1:500; Bioss, Beijing, China), rabbit anti-HO-1 antibody (1:1000; Bioss, Beijing, China), and rabbit anti-β-actin antibody (1:5000; Bioss, Beijing, China) as the internal control. The secondary antibody used was HRP-conjugated goat anti-rabbit IgG (1:10,000; Proteintech, Wuhan, China).

RT–PCR via dye-based detection was performed as described in Section 2.4. The primer sequences are shown in Table 1.

The steps for indirect immunofluorescence were as follows: when the PK-15 cells reached confluency, 0.03125 mg/mL rosmarinic acid was mixed with an equal volume of 100 TCID50 PRV solution and incubated at 37 °C for 1 h. The above mixture was added to a 96-well cell culture plate and incubated for 1 h. After PBS washes, cell maintenance medium was added. After 36 h, the samples were fixed with 4% paraformaldehyde. The samples were washed with PBS to remove any residual fixative. The cells were permeabilized with 0.1% Triton X-100 to allow the antibodies to enter the intracellular space. After permeabilization, the samples were washed again with PBS. The cells were incubated with blocking solution (PBS containing 2% serum) for 1 h. Nrf2 antibody (1:200) was added to the cells. The mixture was gently mixed and incubated overnight at 4 °C. The cells were washed three times with PBS to remove unbound Nrf2 antibody. The samples were incubated with a fluorescently labelled secondary antibody (1:500; Proteintech, Wuhan, China) for 1 h at room temperature on a shaker protected from light. The samples were washed three times with PBS. Then, PBS solution containing DAPI was added to the cells, which were incubated at room temperature for 5–10 min. The cells were washed three more times with PBS. The samples were observed under a fluorescence microscope, and images were captured. To quantify Nrf2 nuclear translocation, immunofluorescence images were analyzed for fluorescence intensity using ImageJ-1.53. For each image, the DAPI and Nrf2 channels were separated, and the mean Nrf2 fluorescence was measured in the nuclear region and in an equivalent cytoplasmic region. The nuclear-to-cytoplasmic (N/C) ratio of Nrf2 fluorescence intensity was then calculated.

### 2.9. Statistical Analysis

All the cell experiments were performed in triplicate, and the results are presented as the means ± standard deviations. The statistical significance of the data was evaluated via a two-tailed Student’s *t* test in GraphPad Prism 8.4 software (La Jolla, CA, USA). A *p* value less than 0.05 was considered statistically significant (ns: *p* > 0.05, * *p* < 0.05, ** *p* < 0.01, *** *p* < 0.001, **** *p* < 0.0001, # *p* < 0.05, ## *p* < 0.01, ### *p* < 0.001, and #### *p* < 0.0001).

## 3. Results

### 3.1. Rosmarinic Acid Inhibits the Replication of PRV in PK-15 Cells

**Figure 1:**
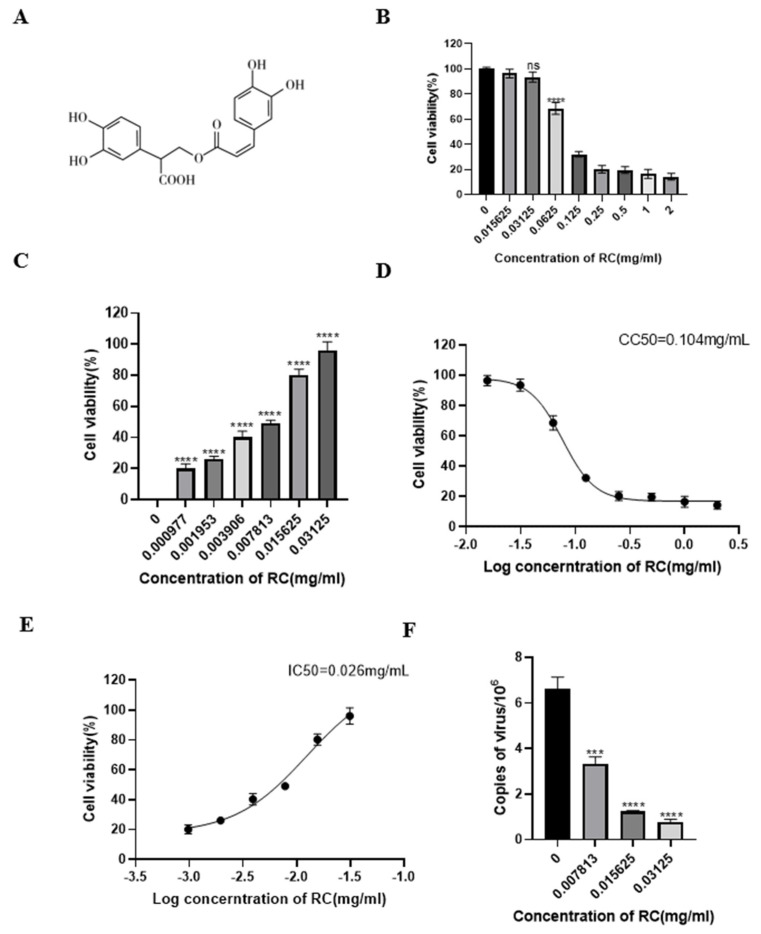
Shows that rosmarinic acid inhibited the proliferation of PRV in PK-15 cells. (**A**) Chemical structure of rosmarinic acid. (**B**) A CCK-8 assay was used to determine the cytotoxicity of rosmarinic acid to PK-15 cells at concentrations ranging from 0.0015625 to 2 mg/mL. The cell viability is expressed as a percentage of the control group cell activity. (**C**) The inhibition rate of rosmarinic acid against PRV. The CCK-8 assay was used to determine the viability of PK-15 cells to calculate the inhibitory effect of rosmarinic acid on PRV. The inhibition rate (%) = (OD450 value of the herbal medicine test group − OD450 value of the virus control group)/(OD450 value of the cell control group − OD450 value of the virus control group) × 100%. (**D**) CC50 of rosmarinic acid in PK-15 cells. (**E**) The IC50 of rosmarinic acid for inhibiting the replication of PRV in PK-15 cells. (**F**) FQ–PCR analysis revealed that rosmarinic acid reduced the number of viral DNA copies in PRV-infected cells. Compared with the control group without rosmarinic acid treatment, ns indicates *p* > 0.05, *** indicates *p* < 0.001, and **** indicates *p* < 0.0001.

In this study, rosmarinic acid was diluted in DMEM such that the final DMSO concentration was less than 1%, which does not affect the virus or cells. The cytotoxicity of rosmarinic acid was assessed by evaluating the viability of PK-15 cells via microscopic examination and CCK-8 assays. The results revealed that as the concentration of rosmarinic acid increased, cell viability gradually decreased. When the concentration of rosmarinic acid was less than 0.03125 mg/mL, the viability of the PK-15 cells was not significantly different from that of the control cells (Figure 1B). Therefore, this concentration was selected as the safe concentration for subsequent cell experiments. The CC50 of rosmarinic acid was calculated to be 0.1043 mg/mL.

The inhibitory activity of rosmarinic acid against PRV in PK-15 cells was evaluated via the CPE, CCK-8, and FQ–PCR methods. The CCK-8 results revealed that rosmarinic acid significantly inhibited PRV infection-induced cell death in a dose-dependent manner. When the concentration of rosmarinic acid was 0.03125 mg/mL, the inhibition rate was as high as 98.78%. Even at a concentration of 0.007813 mg/mL, the inhibition rate against PRV was still greater than 50% (Figure 1C). The calculated IC50 of rosmarinic acid was 0.02654 mg/mL, and the selectivity index (SI, CC50/IC50) was 3.9. In the viral copy number determination experiment, the viral copy number was reduced in a dose-dependent manner in the presence of rosmarinic acid. At rosmarinic acid concentrations of 0.03125 mg/mL, 0.015625 mg/mL, and 0.007813 mg/mL, the viral copy numbers decreased by 88.19%, 81.34%, and 49.31%, respectively (Figure 1D). Overall, these results indicate that rosmarinic acid can significantly inhibit the replication of PRV in PK-15 cells.

### 3.2. Rosmarinic Acid Affects Specific Stages of the PRV Life Cycle

**Figure 2:**
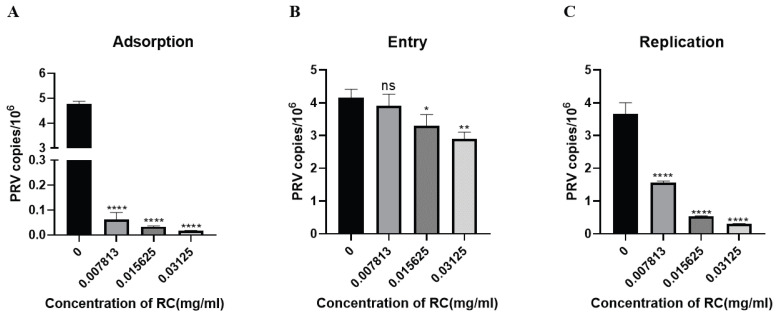
Illustrates the mechanism by which rosmarinic acid inhibits PRV. (**A**) Rosmarinic acid inhibited the PRV adsorption process. (**B**) Rosmarinic acid inhibited the PRV entry process. (**C**) Rosmarinic acid inhibited the intracellular replication of PRV. (Compared with the untreated control group, ns indicates *p* > 0.05, * indicates *p* < 0.05, ** indicates *p* < 0.01, and **** indicates *p* < 0.0001).

For further analysis of the antiviral mechanism of rosmarinic acid in PK-15 cells, real-time fluorescent quantitative PCR was performed via a probe assay to measure the viral copy number in infected cells at different stages of the PRV life cycle. In the adsorption experiment, when rosmarinic acid and PRV were added simultaneously to the cells at 4 °C, rosmarinic acid significantly reduced the copy number of viral DNA. In the 0.03125 mg/mL treatment group, the PRV copy number was 99.66% lower than that in the untreated group, indicating that rosmarinic acid inhibited the PRV attachment process (Figure 2A). In the experimental setup, the virus was allowed to infect the cells at 4 °C. The cells were then treated with rosmarinic acid. Compared with the untreated group, the treatment group with the highest concentration of rosmarinic acid presented a 30.11% reduction in the PRV copy number (Figure 2B). After the virus enters the host cell, it begins to utilize the raw materials and energy systems provided by the host cell to replicate itself. In the replication assay, we found that rosmarinic acid significantly inhibited the replication of PRV. At a rosmarinic acid concentration of 0.03125 mg/mL, the viral copy number decreased by 91.55% (Figure 2C).

### 3.3. Rosmarinic Acid Reduces the Expression of the PRV gB Protein

**Figure 3:**
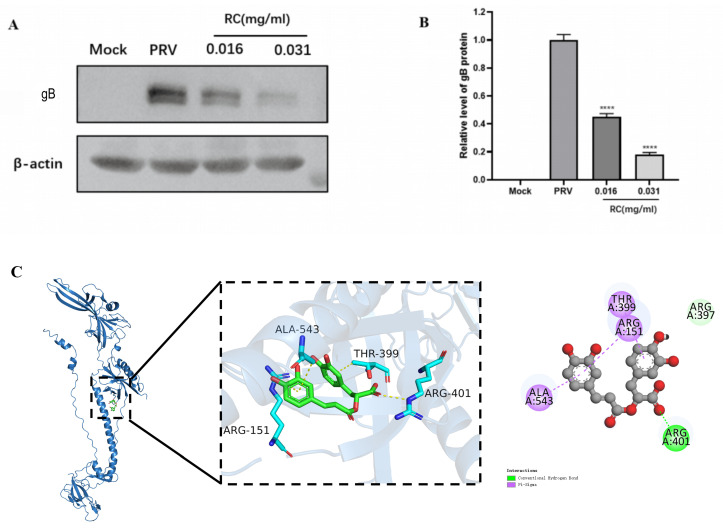
Rosmarinic acid inhibits the expression of the PRV gB protein. (**A**) The relative expression of the gB protein. (**B**) The grayscale intensity of the gB protein was quantified. (**C**) Optimal binding between the PRV gB protein and the rosmarinic acid molecule. The green rod-like structure represents rosmarinic acid, and the PRV gB protein is represented by a cartoon. The key amino acid residues are shown as sticks and labelled, and the hydrogen bonds formed between rosmarinic acid and the gB amino acids are indicated by yellow dashed lines. (Compared with PRV, **** indicates *p* < 0.0001).

Rosmarinic acid significantly inhibited the expression level of the PRV gB protein (Figure 3A). On the gB receptor, Arg401 formed a hydrogen bond with rosmarinic acid, while Arg151, Thr399, and Ala543 engaged in hydrophobic interactions with the ligand (Figure 3C). The binding energy was −6.7 kcal/mol, which indicated that rosmarinic acid forms a stable complex with the PRV gB protein.

### 3.4. Rosmarinic Acid Inhibits PRV Infection in Mice

**Figure 4:**
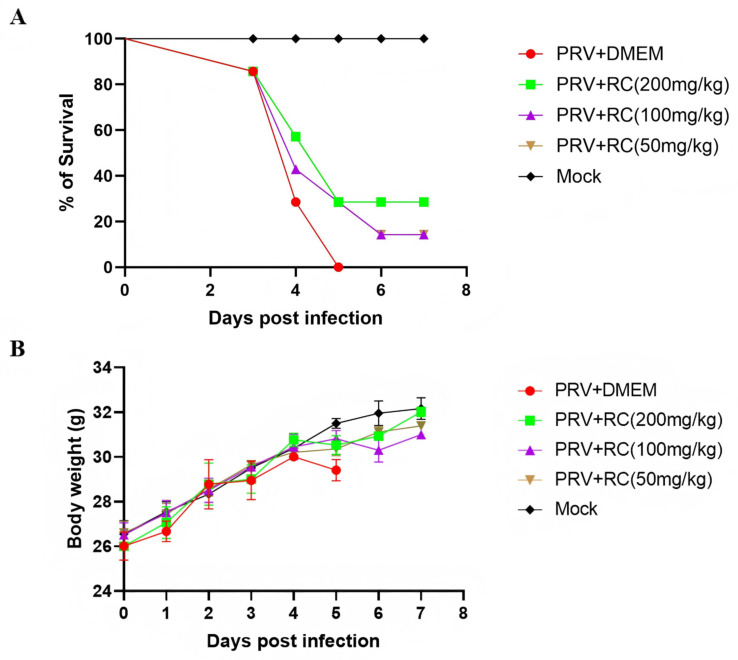

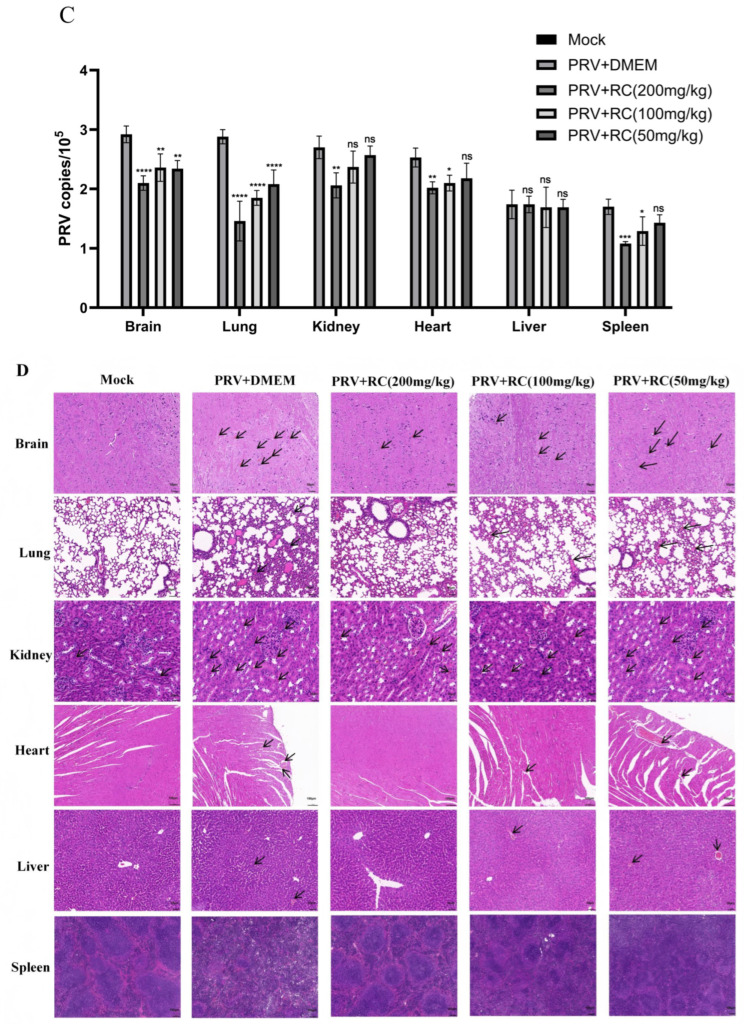
In vivo inhibition of PRV replication by rosmarinic acid. (**A**) The survival rates for each group of mice were calculated via the following formula: survival rate = number of surviving mice/total number of mice. (**B**) Changes in the body weights of the mice in each group. (**C**) Viral loads in the organ tissues of the mice in the different groups. On day 3 post-PRV challenge, the brain, lung, kidney, heart, liver, and spleen tissues of the mice were collected, and viral DNA was extracted. The viral copy numbers in the organ tissues were then detected via FQ–PCR. (**D**) Pathological sections of the brain, lung, kidney, heart, liver, and spleen tissues of the mice. The black arrows indicate the main pathological changes in the sections. (Compared with the PRV control group, ns indicates *p* > 0.05, * indicates *p* < 0.05, ** indicates *p* < 0.01, *** indicates *p* < 0.001, **** indicates *p* < 0.0001).

In the virus challenge control group, the mice began to exhibit restlessness, pruritus, convulsions, and moist skin with scratching, erosion, and bleeding at the inoculation site starting on day 3 post-challenge. All the mice in this group died by day 5, resulting in a 0% survival rate. In the low-dose rosmarinic acid group, the mice began to show clinical symptoms starting on day 3 post- challenge, and death started. By day 7, only 1 mouse remained alive, resulting in a survival rate of 14.3%. In the medium-dose group, severe clinical symptoms were observed on day 4, and the final survival rate was 14.3%. In the high-dose rosmarinic acid group, the mice presented clinical symptoms and mortality on day 4 post-challenge, with a survival rate of 28.5%. All the mice in the normal control group without challenge survived. The results revealed that rosmarinic acid treatment delayed the onset of clinical symptoms, prolonged the average survival time, and increased the survival rate of PRV-infected mice (Figure 4A). The body weight change curve of the mice revealed that, compared with the normal control group, the challenged control group presented a slower growth rate after the onset of symptoms on day 2 post-challenge and a decrease in body weight by day 5 post-challenge. In the rosmarinic acid treatment group, the average body weight of the mice slightly decreased on days 4 and 5 but then tended to increase after day 6 (Figure 4B).

Changes in the viral copy numbers in the brain, lung, kidney, heart, liver, and spleen tissues of the mice were detected via FQ–PCR. The results revealed that in the challenge control group, PRV was detected in the brain, lung, kidney, heart, liver, and spleen tissues of the mice, with the highest viral load found in the brain tissue. Compared with the challenge control, rosmarinic acid at 200 mg/kg, 100 mg/kg, and 50 mg/kg was able to reduce the viral load in the brain and lung tissues. In the high-dose rosmarinic acid group (200 mg/kg), the best efficacy was observed, but there was no significant effect on the viral load in the liver tissue.

For further analysis of the protective effect of rosmarinic acid on the damage caused by PRV infection, histopathological examination was performed on the brain, lung, kidney, heart, liver, and spleen tissues collected from the various groups of mice. As shown in Figure 4D, the HE staining results indicated that after viral infection, mild microglial proliferation accompanied by neuronal degeneration and necrosis in the brain. Lung sections revealed eosinophilic serous exudates within alveolar spaces, with thickened alveolar septa and mild infiltration of neutrophils and mononuclear cells. Scattered lymphocytes and macrophages were observed in the renal cortex. The myocardium exhibited myofiber disruption, disorganization, and focal hemorrhage. In the liver, mild lymphocytic infiltration and sinusoidal congestion were noted. The spleen displayed an indistinct boundary between white and red pulp, along with a reduction in red pulp. After rosmarinic acid treatment, the pathological changes in the tissue were alleviated.

### 3.5. Changes in Cytokine Levels

**Figure 5:**
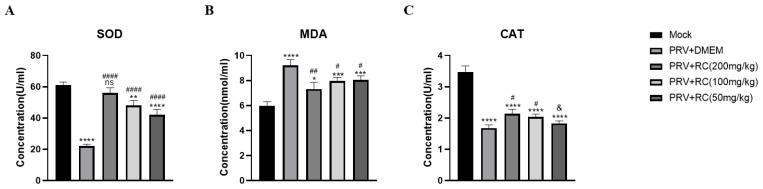
Concentrations of SOD (**A**), MDA (**B**), and CAT (**C**) in the serum of mice from different groups. On day 3 post-PRV infection, three mice were randomly selected from each group, and blood samples were collected to measure the levels of four cytokines in the mouse serum. (Compared with the cell control group, ns indicates *p* > 0.05, * indicates *p* < 0.05, ** indicates *p* < 0.01, *** indicates *p* < 0.001, **** indicates *p* < 0.0001; compared with the virus control group, & indicates *p* > 0.05, # indicates *p* < 0.05, ## indicates *p* < 0.01, #### indicates *p* < 0.0001).

To further investigate whether rosmarinic acid protects against PRV infection through an antioxidant pathway, we determined the levels of oxidative stress-related cytokines in the serum of PRV-infected mice. Serum was collected from the mice in each group, and an ELISA kit was used to identify changes in the levels of the cytokines SOD, MDA, and CAT in the mouse serum. As shown in Figure 5, compared with the normal control, PRV infection significantly increased the serum levels of the cytokine MDA (*p* < 0.05) and significantly decreased the levels of the cytokines SOD and CAT in the mice. Compared with the virus control group, the rosmarinic acid treatment group presented significantly elevated levels of the antioxidant enzymes SOD and CAT in the mouse serum (*p* < 0.05). In the high-dose SOD group, the levels were increased until they were not significantly different from those in the normal control group. Additionally, the rosmarinic acid treatment group presented significantly decreased levels of the oxidative stress marker MDA in the mouse serum (*p* < 0.05).

### 3.6. Network Pharmacology Study of the Antioxidant Effects of Rosmarinic Acid

#### 3.6.1. Screening of the Antioxidant Targets of Rosmarinic Acid

Through the PharmMapper database, 854 potential targets of rosmarinic acid were identified. Additionally, 1150 antioxidant targets were identified from the GeneCards database. By taking the intersection of rosmarinic acid and antioxidant targets, we identified 74 potential targets for the antioxidant effects of rosmarinic acid.

#### 3.6.2. Identification of Antioxidant Targets of Rosmarinic Acid by Venn Diagram Analysis

**Figure 6:**
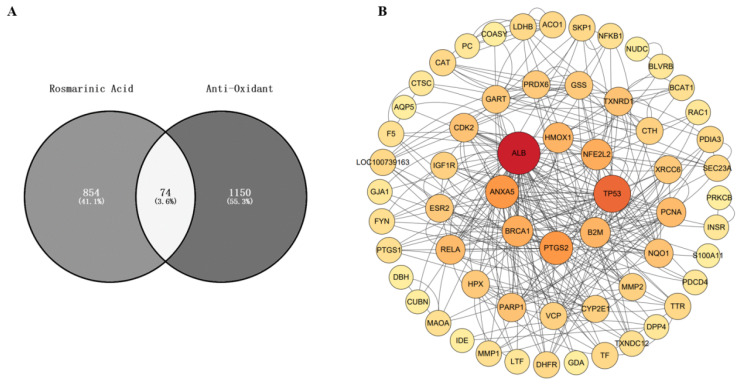
Network pharmacology analysis of the antioxidant mechanism of RC. (**A**) Analysis of the antioxidant targets of rosmarinic acid. With the online Venny 2.1 software tool, a Venn diagram was generated to visualize the antioxidant targets of rosmarinic acid. (**B**) Cytoscape network pharmacology analysis based on topological methods. The relationship of the key genes; the intensity of the colour indicates the magnitude of the degree value.

The results were logged into the online bioinformatic visualization platform, and the antioxidant targets and the targets of rosmarinic acid were input into the proportional Venn diagram of disease targets and drug targets, respectively. The antioxidant targets of rosmarinic acid were visualized via a Venn diagram (Figure 6A).

#### 3.6.3. Constructing a PPI Network

A total of 74 potential antioxidant targets of rosmarinic acid were screened and input into the String database to construct a PPI network (Figure 6B). The network had 74 nodes and 153 edges, with an expected number of edges of 54. The average node degree was 4.14, and the average local clustering coefficient was 0.446. The PPI enrichment *p* value was <1.0 × 10^−16^. The network data were saved as TSV files and imported into Cytoscape 3.8.2 software. A topological analysis of the network was conducted via the Network Analyzer tool. Nodes with a degree greater than twice the median degree (20) were selected as the filtering criterion. The median betweenness centrality (0.0404) and median closeness centrality (0.7167) were used as the selection criteria. A HitHub network was constructed on the basis of the selection criteria mentioned above, and the core target genes were screened. The results indicated that the core antioxidant targets of rosmarinic acid are ALB, TP53, ANXA5, PTGS2, and NFE2L2.

#### 3.6.4. Functional Enrichment Analysis via GO and Pathway Analysis via KEGG

**Figure 7:**
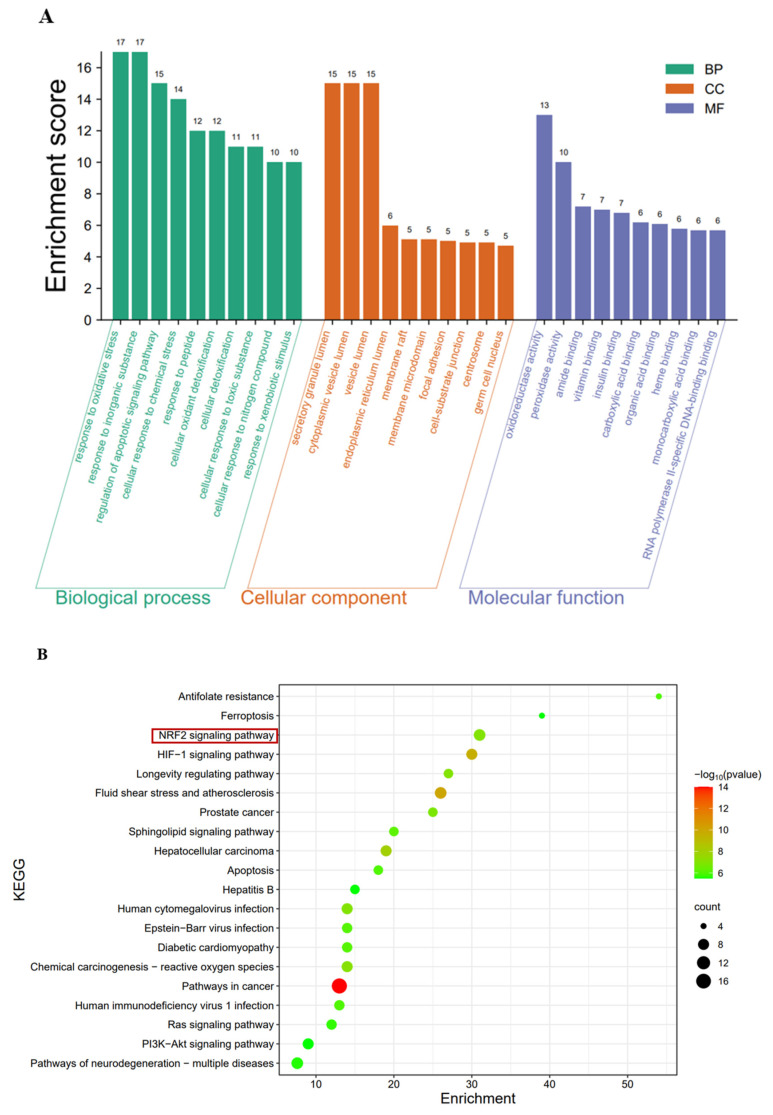
Potential pathways regulated by RC. (**A**) Functional enrichment analysis of key genes in the RC pathway. (**B**) KEGG pathway enrichment analysis bubble plot. The signaling pathways selected for subsequent studies are highlighted with red boxes.

When 74 rosmarinic acid antioxidant targets were input into the Metascape database, GO functional analysis was performed. After the GO functional enrichment analysis, the top 10 results ranked by *p* value for each term were selected. As shown in Figure 7A, the results revealed that the biological process (BP) category included 536 terms (*p* < 0.01). The main groups included the response to oxidative stress, the response to inorganic substances, the regulation of apoptosis signaling pathways, the cellular response to chemical stress, the response to peptides, the cellular detoxification of oxidants, the cellular efflux of toxins, the response to toxic substances, the cellular response to abiotic stimuli, and the cellular response to environmental stimuli. The molecular function (MF) category included 66 terms (*p* < 0.01). The main terms were oxidoreductases, peroxidases, amide binding, vitamin binding, insulin binding, carboxylic acid binding, organic acid binding, haem binding, monocarboxylic acid binding, and the binding of RNA polymerase II-specific DNA-binding transcription factors. The cellular component (CC) contained 54 terms (*p* < 0.01). The main terms were the secretory granule lumen, cytoplasmic vesicle lumen, vesicle lumen, endoplasmic reticulum lumen, membrane rafts, membrane microdomains, focal adhesions, battery substrate connections, centrosomes, and reproductive cell nuclei.

The KEGG analysis identified 108 relevant signaling pathways (*p* < 0.01). The main pathways involved include cancer signaling pathways, sphingolipid signaling pathways, and Epstein–Barr virus (EBV) infection. According to the *p* value, the top 20 pathways were selected and plotted in a bubble chart, as shown in Figure 7B. Three pathways are associated with the antioxidant response: the Nrf2 signaling pathway, the chemical carcinogenesis-reactive oxygen species pathway, and the ferroptosis pathway. Additionally, three pathways were associated with viral infection, namely, the human immunodeficiency virus type 1 infection pathway, the cytomegalovirus infection pathway, and the Epstein–Barr virus infection pathway.

### 3.7. Rosmarinic Acid Activates the Nrf2 Signaling Pathway in PRV-Infected Cells

**Figure 8:**
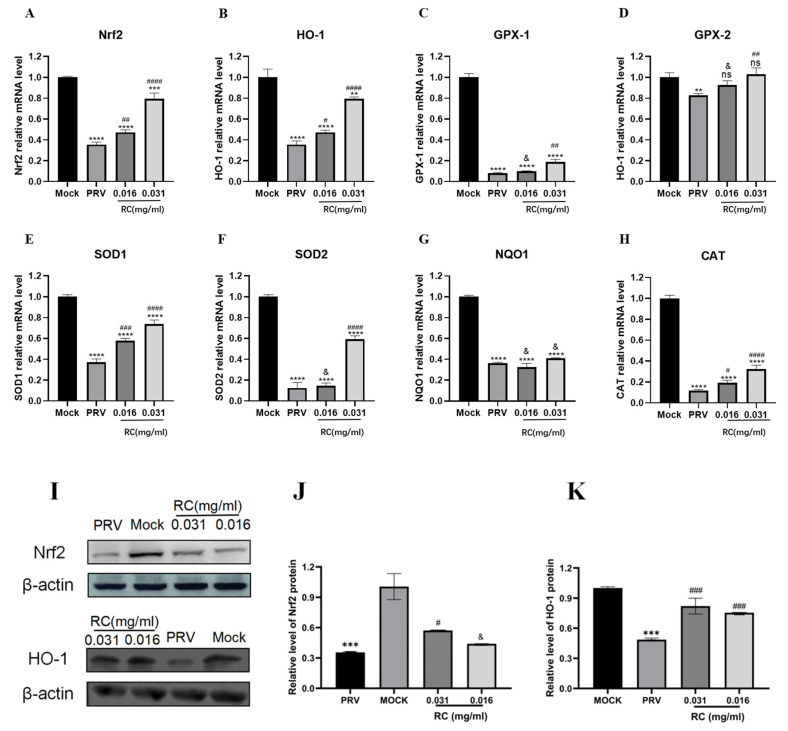

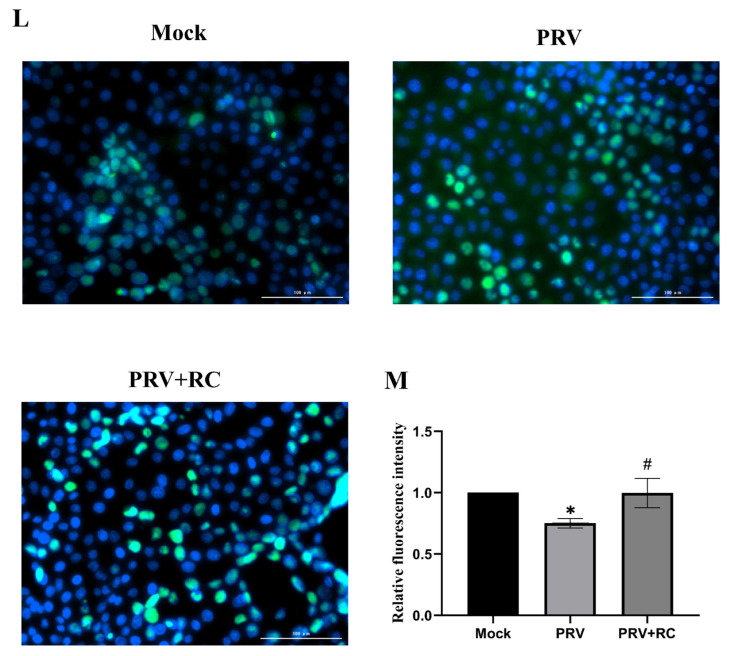
Effects of rosmarinic acid on the Nrf2 signaling pathway. (**A**): Transcriptional level of the Nrf2 gene. (**B**): Transcriptional level of the HO-1 gene. (**C**): Transcriptional level of the GPX-1 gene. (**D**): Transcriptional level of the GPX-2 gene. (**E**): Transcriptional level of the SOD1 gene. (**F**): Transcriptional level of the SOD2 gene. (**G**): Transcriptional level of the NQO1 gene. (**H**): Transcriptional level of the CAT gene. (**I**): Effect of rosmarinic acid on the Nrf2 and HO-1 proteins. (**J**): Quantification of grayscale values for the Nrf2 protein shown in (**I**). (**K**): Quantification of grayscale values for the HO-1 protein shown in (**I**). (**L**): Nuclear translocation of the Nrf2 protein is shown, where blue fluorescence represents the cell nucleus and green fluorescence represents the Nrf2 protein. (**M**): Semi-quantitative analysis of (**J**). (Compared with the cell control group, ns indicates *p* > 0.05, * indicates *p* < 0.05, ** indicates *p* < 0.01, *** indicates *p* < 0.001, **** indicates *p* < 0.0001; compared with the virus control group, & indicates *p* > 0.05, # indicates *p* < 0.05, ## indicates *p* < 0.01, ### indicates *p* < 0.001, #### indicates *p* < 0.0001).

The Nrf2 signaling pathway, an antioxidant cellular pathway, primarily participates in the antioxidant response through the expression of antioxidant genes. To verify the effect of rosmarinic acid on the Nrf2 pathway in PRV-infected cells, we used real-time quantitative PCR to detect the transcriptional changes in Nrf2, HO-1, and downstream antioxidant genes (GPX1, GPX2, SOD1, SOD2, NQO1, and CAT) in the Nrf2 signaling pathway. The real-time quantitative PCR results revealed that, compared with the virus control, rosmarinic acid significantly increased the transcription levels of the Nrf2, HO-1, GPX1, GPX2, SOD1, SOD2, and CAT genes in the infected cells (*p* < 0.05). Compared with the control conditions, PRV infection resulted in a significant decrease (*p* < 0.05) in the transcript levels of the Nrf2, HO-1, GPX1, GPX2, SOD1, SOD2, NQO1, and CAT genes, as shown in Figure 8A–H. The results revealed that PRV infection suppressed the Nrf2 signaling pathway and the transcription of downstream antioxidant factors, whereas rosmarinic acid increased the activation of the Nrf2 signaling pathway and the transcription of antioxidant factors.

Western blot analysis was used to determine the protein expression level of Nrf2 in PRV-infected cells at 36 h post-infection. The grayscale value analysis was performed via ImageJ software. Compared with the virus control, rosmarinic acid at concentrations of 0.031 mg/mL significantly increased the protein expression levels of Nrf2 and HO-1 (*p* < 0.05). Compared with those in the control group, the protein expression levels of Nrf2 and HO-1 were significantly lower (*p* < 0.05) in the virus control group. As shown in Figure 8I–K, PRV infection suppressed the expression of the cellular Nrf2 and HO-1 proteins, whereas rosmarinic acid significantly activated the PRV infection-induced activation of the Nrf2 signaling pathway.

Immunofluorescence microscopy revealed that the Nrf2 fluorescence signal was predominantly localized in the cell nucleir of the rosmarinic acid-treated group, whereas it was mainly observed in the cytoplasm of the virus control group. Treatment with rosmarinic acid promoted the translocation of Nrf2 from the cytoplasm to the nucleus, as shown in Figure 8L.The immunofluorescence images were semi-quantitatively analyzed with Image J (Figure 8M). Relative to the blank control, nuclear Nrf2 levels were significantly reduced in the virus control group (*p* < 0.05). Conversely, rosmarinic acid treatment significantly increased nuclear Nrf2 content compared with the virus control (*p* < 0.05).

## 4. Discussion

PR primarily occurs in domestic pigs but can also occur in wild boars, canids, felids, and rodents [20] and can even infect humans [6]. PRV spreads rapidly in swine herds, leading to various clinical symptoms. After infection, treatment is relatively difficult, resulting in high mortality rates [5]. Currently, there are no effective specific drugs to treat PR in pigs [21], and the emergence of vaccine-escaping variants further underscores this clinical need [22,23].

In this context, natural products like rosmarinic acid, a polyphenol with documented antioxidant and antiviral activities [14,15,16,17], represent promising candidates. This study investigated the antiviral activity of rosmarinic acid against PRV. The results revealed that rosmarinic acid exhibited significant inhibitory activity against PRV in PK-15 cells. The SI value was 3.9, indicating that the drug had significant bioactivity at a concentration of 0.03125 mg/mL.This finding confirms rosmarinic acid’s potential for further development.

To delineate rosmarinic acid ‘s mechanism of action, we first investigated its impact on the PRV life cycle. This study demonstrated that rosmarinic acid can significantly reduce viral DNA copy numbers and inhibit viral replication at multiple stages, including viral adsorption, cell entry, and intracellular replication. Notably, rosmarinic acid resulted in a remarkable 99.66% reduction in viral adsorption. Furthermore, we confirmed that rosmarinic acid significantly suppresses the expression of the glycoprotein B, a protein crucial for viral entry and cell-to-cell spread, an effect shared by other natural antivirals like luteolin [11]. This multi-stage inhibition underscores rosmarinic acid’s potential as a robust antiviral agent.

Rosmarinic acid can reduce the mortality rate of JEV-infected mice. In JEV-infected animals treated with rosmarinic acid, the viral load and proinflammatory cytokine levels are significantly decreased [18]. The therapeutic potential of rosmarinic acid was further validated in a murine model of PRV infection. The results demonstrated that the administration of rosmarinic acid before and after challenge conferred significant protective effects against PRV infection in the mice. In the challenge control group, the mice rapidly developed neurological symptoms and experienced 100% mortality following infection. In contrast, the low-, medium-, and high-dose rosmarinic acid groups all presented varying degrees of prolonged survival and reduced mortality rates. The viral load is a direct parameter for evaluating the antiviral effect in vivo, as it can reflect the replication of the virus in different organs [24,25]. Rosmarinic acid significantly reduced the copy numbers of PRV in the brain, lung, kidney, heart, and spleen tissues of the mice. In the HE-stained sections, the pathological changes in various organs were alleviated after rosmarinic acid treatment. Since oxidative stress is a key contributor to viral pathogenesis [26,27], and given rosmarinic acid’s established antioxidant profile, we investigated its impact on the systemic redox state. In the PRV-infected mice, the serum levels of MDA were significantly increased, whereas the levels of SOD and CAT were significantly decreased. These findings indicate that infection causes oxidative stress, leading to increased cell membrane damage and an increased inflammatory response. In the rosmarinic acid treatment groups, the levels of SOD and CAT were significantly elevated. In the high-dose group, their levels were not significantly different from those in the normal control group. These results indicate that rosmarinic acid can increase antioxidant enzyme activity and alleviate the oxidative stress induced by PRV infection by modulating the intracellular redox state. Furthermore, the significant reduction in MDA levels further supported the antioxidant properties of the compound, indicating its ability to effectively reduce lipid peroxidation and thereby alleviate cellular damage. This compelling in vivo evidence positioned the Nrf2-mediated antioxidant pathway as a prime candidate for mediating rosmarinic acid’s effects.

To systematically uncover the mechanistic basis of rosmarinic acid’s antioxidant and antiviral effects, we employed a network pharmacology approach. Through the intersection analysis of the PharmMapper and GeneCards databases, 74 potential antioxidant targets of rosmarinic acid were identified. The PPI network constructed via the STRING database revealed the complex relationships among 74 identified targets. PPI enrichment analysis revealed the statistical significance of the network. The core target genes are ALB, TP53, ANXA5, PTGS2, and NFE2L2. ALB is the main colloid osmotic pressure-regulating factor in plasma, helping to maintain the liquid balance in the blood. This molecule can also bind to free radicals, exerting an antioxidant effect [28,29]. TP53 is a tumour suppressor protein that can activate DNA repair mechanisms, helping cells repair damaged DNA [30]. ANXA5 is a calcium-dependent membrane-binding protein that can exert its effects through the Nrf2 pathway in DBP-induced oxidative stress [31]. PTGS2 catalyses the conversion of arachidonic acid to prostaglandins and other bioactive molecules, which can attenuate coronary atherosclerosis [32]. NFE2L2, also known as Nrf2, is a ubiquitous and evolutionarily conserved intracellular defence mechanism that functions to counteract oxidative stress [33]. The main actions of rosmarinic acid include antioxidant activity, cancer prevention, and metabolic regulation [34,35]. The results of the GO functional analysis revealed that rosmarinic acid affects various biological processes, particularly in response to oxidative stress, cell apoptosis, and chemical stimuli. The enrichment of these functions suggested that rosmarinic acid promoted the antioxidant capacity of cells by modulating multiple signaling pathways. KEGG pathway analysis revealed that rosmarinic acid affects signaling pathways involved in cancer, sphingolipid metabolism, and viral infection, with a particular emphasis on the Nrf2 signaling pathway, highlighting its importance in regulating the antioxidant response. This bioinformatic prediction provided a strong foundation for our subsequent experimental validation, squarely focusing our investigation on the Nrf2 pathway.

The Nrf2 (NFE2L2) pathway plays crucial roles in the cellular response to oxidative stress, immune regulation [36], diabetes [37], and cancer prevention and treatment [38,39] and is central to redox homeostasis in animal cells [40]. For example, glycyrrhizin and sulforaphane, have been shown to exert their antiviral activities through Nrf2 pathway activation [41]. These precedents supported our hypothesis that rosmarinic acid might also engage this pathway to combat PRV infection.

Nrf2 is a key regulatory factor for the defence of cells against oxidative stress, and its activation can promote the expression of antioxidant genes, thereby reducing oxidative damage [33]. Studies have shown that *Costus speciosus* extract can prevent ZEN-induced oxidative damage by regulating the expression of the Nrf2 gene [42]. To verify whether rosmarinic acid alleviates PRV infection by mediating the protective effects on cells through the Nrf2 pathway, we determined the expression levels of Nrf2 pathway-related proteins and downstream antioxidant genes. We found that PRV infection itself suppressed the Nrf2 pathway, as shown by decreased protein levels of Nrf2 and HO-1, along with reduced transcription of antioxidant genes (such as GPX1, GPX2, SOD1, SOD2, and CAT). Treatment with rosmarinic acid robustly reversed this suppression, significantly upregulating the expression of Nrf2, HO-1, and a suite of antioxidant genes. When cells are under oxidative stress or other stimuli, Nrf2 translocates from the cytoplasm to the nucleus. The activation of the Nrf2 pathway is contingent upon its translocation to the nucleus. Eicosapentaenoic acid (EPA) can promote the nuclear translocation of Nrf2 to exert antioxidant effects, thereby suppressing endoplasmic reticulum stress and alleviating senescence [42]. Polyphenols from honeysuckle (HPs) can effectively occupy the Kelch homologue (Kelch) structural domain of Keap1, competitively binding with Nrf2, ultimately increasing the translocation of Nrf2 into the cell nucleus and repairing cellular damage [43]. Porcine reproductive and respiratory syndrome virus (PRRSV) reduces cellular antioxidant capacity by promoting Keap1-dependent ubiquitination and degradation of Nrf2, thereby facilitating evasion of host antiviral immunity [44]. Nrf2 also activates antiviral responses through pattern-recognition receptor pathways such as RIG-I/MAVS. For example, Sec10 deficiency enhances innate immune activity and reduces viral load via the NRF2–ATF4–RIG-I axis [45]. Compared with the virus control group, the rosmarinic acid treatment group showed increased nuclear translocation of Nrf2. In conclusion, our findings delineate a novel mechanism whereby rosmarinic acid activates the Nrf2-mediated antioxidant defense system to counteract PRV infection.

Integrating the above results, we preliminarily conclude that rosmarinic acid inhibits PRV through two complementary mechanisms: direct antiviral activity and activation of the Nrf2 pathway to alleviate oxidative stress. These mechanisms likely act synergistically; by lowering viral load, the direct antiviral effect reduces the immediate burden of infection, while the antioxidant response remodels the intracellular environment in ways that are unfavorable to viral replication, together mediating anti-PRV activity. Hu et al. [46] demonstrated that rosmarinic acid also activates the cGAS-STING signaling pathway to stimulate innate immunity, increases expression of the downstream antiviral effector IFN-β, and reduces inflammation and apoptosis in PRV-infected cells, thereby producing additional anti-PRV effects. Taken together with the present study, these findings indicate that rosmarinic acid concurrently targets the host antioxidant defense system and the innate immune system; this multi-target profile confers distinct advantages in modulating virus–host interactions. Because PRV establishes latency in the trigeminal and dorsal root ganglia and can reactivate to cause disease under stress, immunosuppression, or other stimuli—a major obstacle to PR control—future studies should examine whether rosmarinic acid’s enhancement of host defenses affects viral latency and reactivation. Such work would provide a scientific basis for developing integrated strategies aimed at eradicating PRV infection.

## 5. Conclusions

Collectively, our findings unveil that rosmarinic acid is a promising anti-PRV agent. Rosmarinic acid exerts its antiviral effect through a dual mechanism: it directly targets the viral life cycle by inhibiting viral adsorption, entry, and intracellular replication, and indirectly by activating the host Nrf2 signaling pathway to counter virus-induced oxidative stress. These findings not only identify rosmarinic acid as a promising natural lead compound for the development of anti-PRV agents but also provide a novel pharmacological strategy that combines direct antiviral activity with enhanced host cellular defense mechanisms.

## Figures and Tables

**Table 1:** Sequences of primers used in the Nrf2 signaling pathway assay.

Gene Name	Sequence (5′-3′)
Nrf2-F	CACCACCTCAGGGTAATA
Nrf2-R	GCGGCTTGAATGTTTGTC
HO-1-F	AGGCTGAGAATGCCGAGTTC
HO-1-R	TGTGGTACAAGGACGCCATC
GPX1-F	TGAATGGCGCAAATGCTCAC
GPX1-R	GCTTCGATGTCAGGCTCGAT
GPX2-F	TTGCCAAGTCCTTCTACGA
GPX2-R	GAAGCCAAGAACCACCAG
SOD1-F	GAGACCTGGGCAATGTGACT
SOD1-R	CTGCCCAAGTCATCTGGTTT
SOD2-F	TGGAGGCCACATCAATCATA
SOD2-R	AGCGGTCAACTTCTCCTTGA
NQO1-F	CCAGCAGCCCGGCCAATCTG
NQO1-R	AGGTCCGACACGGCGACCTC
CAT-F	ACGCCTGTGTGAGAACATTG
CAT-R	GTCCAGAAGAGCCTGAATGC
β-actin-F	TGCGGGACATCAAGGAGAA
β-actin-R	AGGAAGGAGGGCTGGAAGA

## Data Availability

The original contributions presented in this study are included in the article. Further inquiries can be directed to the corresponding authors.
